# Tmem45b modulates itch via endoplasmic reticulum calcium regulation

**DOI:** 10.3389/fphys.2025.1708686

**Published:** 2025-12-10

**Authors:** Sa-shuang Wang, Chen Liang, Ruo-lin Wang, Ze-lin Sun, Peng-yu Ren, Bin Wu, Juan-juan Sun, Li Fu, Li-zu Xiao, Wu-ping Sun, Chang-lin Li

**Affiliations:** 1 Department of Pain Medicine and Shenzhen Municipal Key Laboratory for Pain Medicine, Shenzhen Nanshan People’s Hospital, and the 6th Affiliated Hospital of Shenzhen University Medical School, Shenzhen, China; 2 Guangdong Key Laboratory for Biomedical Measurements and Ultrasound Imaging, National-Regional Key Technology Engineering Laboratory for Medical Ultrasound, School of Biomedical Engineering, Shenzhen University Medical School, Shenzhen, China; 3 Guangdong Institute of Intelligence Science and Technology, Zhuhai, Guangdong, China; 4 Guangdong Key Laboratory for Genome Stability and Disease Prevention, Department of Pharmacology and Shenzhen International Cancer Center, Shenzhen University Medical School, Shenzhen University, Shenzhen, Guangdong, China; 5 Institute of Medical Research, Northwestern Polytechnical University, Xi’an, China

**Keywords:** DRG-dorsal root ganglion, itch (pruritus), calcium, endoplasmic reticulum, TMEM45B

## Abstract

**Objective:**

This study aimed to investigate the role of Tmem45b, a gene expressed in itch-associated Dorsal root ganglion (DRG) neurons, in the regulation of itch sensation.

**Methods:**

The expression of Tmem45b was examined in DRG neurons. These neurons included Nppb-, Mrgpra3-, and Mrgprd-positive subtypes, which are known to mediate itch. Behavioral response to various pruritogens including β-alanine, chloroquine, histamine, serotonin, and N-met-LTC4 were assessed on Mrgprd-cre::Tmem45b^flox/flox^ conditional knockout (cKO) mice. Chronic itch was evaluated using both atopic dermatitis-like and dry skin-like mouse models. To investigate intracellular calcium dynamics, calcium imaging was performed on dissociated DRG neurons. Additionally, bulk RNA-seq was conducted on DRG from Tmem45b cKO mice to assess transcriptomic changes. Serca1 expression and the calcium storage capacity of the endoplasmic reticulum (ER) were analyzed following Tmem45b deletion.

**Results:**

Tmem45b was found to be expressed in itch-associated DRG neurons. In Tmem45b cKO mice, scratching behavior was reduced in response to β-alanine but increased in response to chloroquine. Notably, chronic itch was alleviated in Tmem45b-deficient mice. Calcium imaging revealed that Tmem45b cKO impaired calcium responses to β-alanine and allyl isothiocyanate, but not to chloroquine. Mechanistically, Tmem45b deficiency led to a significant downregulation of Serca1, reducing ER calcium storage capacity. Pharmacological inhibition of Serca1 in DRG neurons similarly suppressed intracellular calcium release in response to β-alanine and chloroquine.

**Conclusion:**

Tmem45b plays a critical role in nonhistaminergic itch by regulating ER calcium homeostasis through Serca1. Its deficiency reduces itch behavior and impairs calcium signaling in DRG neurons, suggesting that Tmem45b is a potential therapeutic target for chronic itch.

## Introduction

Itch is a prevalent and uncomfortable sensation that evokes a desire to scratch around the affected skin. Itch, pain, touch, and other somatosensations are primarily detected and relayed by dorsal root ganglia (DRG) neurons (Basbaum et al., 2009; Dong and Dong, 2018; Li et al., 2011). Broadly, itch is categorized as either histamine-dependent (histaminergic) or histamine-independent (nonhistaminergic). Most chronic conditions, such as atopic dermatitis and dry skin, are associated with nonhistaminergic itch (Jeffry et al., 2011). A variety of transient receptor potential (TRP) channels and receptors expressed in DRG neurons are essential for nonhistaminergic itch, such as anoctamin 1, TRPA1, and mas-related G-protein coupled receptor (Mrgprs) family (Kim et al., 2024; Liu et al., 2009; Wilson et al., 2011). The vesicular Zn^2+^ transporter Tmem163 is required for senile pruritus (Tong et al., 2024). However, the treatment for nonhistaminergic itch is still limited.

Traditionally, nonpeptidergic DRG neurons labelled by isolectin B4 (IB4), and peptidergic neurons expressing calcitonin gene-related peptide (CGRP) (Basbaum et al., 2009). Studies have shown that IB4-positive neurons mediate itch responses and mechanical pain (Pinto et al., 2019; Wang et al., 1994; Ye et al., 2012). Based on single-cell RNA sequencing (scRNA-seq), neurons can be classfied into specific types. Non-peptidergic DRG neurons are classified as NP1, NP2 and NP3 in the mouse DRG (Usoskin et al., 2015; Zeisel et al., 2018). Recently, small DRG neurons are classified into Gal-, Nppb-, Th-, Mrgpra3-, and Mrgprd-positive neurons (Li et al., 2016; Wang K. et al., 2021). Mrgpra3^+^ and Mrgprd^+^ neurons are predominantly labelled by IB4. Notably, Mrgprd^+^, Mrgpra3^+^, and Nppb^+^ neurons are identified as distinct itch-sensing neurons. Chloroquine (CQ) activates Mrgpra3 to evoke itch (Liu Q. et al., 2012; Liu et al., 2009). Nppb^+^ neurons detect histamine, allergens, or other stimuli, and transmit itch via the Nppb-gastrin-releasing peptide receptor (GRPR) signaling pathway (Liu et al., 2020; Sun and Chen, 2007). β-alanine activates Mrgprd^+^ neurons, inducing itch. Additionally, Mrgprd^+^ neurons function as polymodal nociceptors, responding to both mechanical stimuli and noxious heat (Cavanaugh et al., 2009; Liu Q. et al., 2012; Rau et al., 2009).

Through scRNA-seq analysis, we found that Transmembrane protein 45b (Tmem45b) was specifically expressed in Mrgprd^+^, Mrgpra3^+^, Th^+^, and Nppb^+^ neurons. Tmem45b is a protein with seven putative transmembrane domains and localized in Golgi fractions. Previous study showed that Tmem45b could function as an interferon-stimulated antiviral factor against RNA virus (Yan et al., 2022). Tmem45b knockout mice exhibit reduced inflammatory and tissue injury-induced mechanical pain (Tanioku et al., 2022). However, research on the role of Tmem45b in sensory perception, especially for itch, is currently lacking. Furthermore, how Tmem45b functions at the Golgi apparatus and the mechanisms by which it influences inflammation pain remain unclear. The Golgi apparatus is closely integrated with the endoplasmic reticulum (ER), receiving proteins that have undergone initial folding and modification in the ER for further processing, including glycosylation and sorting at the trans-Golgi network (TGN). SERCA pumps and ryanodine receptors (RyRs), localized to the ER, are key regulators of intracellular calcium dynamics, controlling calcium release and reuptake within the ER lumen (Guo et al., 2014; Periasamy and Kalyanasundaram, 2007). Dysregulation of Serca-mediated calcium transport has been associated with hypersensitivity and allodynia following nerve injury (Gover et al., 2009; McCallum et al., 2006). For instance, Serca2 downregulation in the rat DRG after chronic constriction injury contributes to ER stress, glial activation, and mechanical allodynia (Li et al., 2022). Intracellular Ca^2+^ release can activate Tmem16a, which regulates pain and itch (Kim et al., 2024). However, whether Golgi-localized Tmem45b plays a regulatory role in intracellular ER calcium homeostasis has not been investigated. This study aim to uncover an unrecognized role of Tmem45b in mediating nonhistaminergic itch, especially for chronic itch. The effects on pain sensation were also compared. Calcium imaging was performed to record the calcium responses of the dissociated DRG neurons to various pruritogens. Furthermore, DRG gene profiles of cKO and wild-type (WT) mice were analyzed, leading to the identification of differentially expressed genes associated with ER calcium regulation. Our findings revealed that Tmem45b deficiency attenuated itch response and disrupted the ER calcium homeostasis.

## Methods

### Animals and genotyping

C57BL/6J mice were used in the experiments, all the animal studies were conducted according to procedures approved by Guangdong Institute of Intelligence Science and Technology Animal Care and Use Committee. The mice used at 7–8 weeks of age were selected. Mrgprd-cre mice are donated from Prof. Jinsong Li (Chinese Academy of Sciences, Institute of Biochemistry and Cell Biology), the mouse information is provided on the website (http://www.sibcb.ac.cn/gtp/search2.jsp?eid=141). To generate CRISPR-Cas9 plasmid for Cre Knockin (KI), sgRNAs of C-terminal of target gene Mrgprd (GGTCTGAAGGGAGCCCAACC) were synthesized, annealed, and ligated to the pX330-mCherry plasmid (Addgene, #98750) which was digested with Bbs I (Thermo). For the construction of Cre KI DNA donor, the sequences encoding left homologous arm, T2A-SV40 NLS-Cre and right homologous arm were amplified and ligated to the linear pMD19T vector with 20 bp overlap in order by Seamless Cloning Kit (Beyotime, D7010S). The Tmem45b-flox mice were purchased from the European Mouse Mutant Archive (EMMA ID: 05499). All animals used in the study were male unless otherwise specified as female.

### RNA extraction and real-time PCR

The total RNA was harvested from the fresh tissue and extracted using TRIzol reagent (Thermo Fisher Scientific). 1 μg RNA was reverse transcribed to cDNA with PrimeScript RT Master Mix (Takara). The procedure was as follows: Real-time quantitative PCR (qPCR) was performed with 2x RealStar Fast SYBR Mix (GenStar) and a Bio-Rad CFX Real Time PCR machine. The thermal cycling conditions were 95 °C for 2 min, followed by 40 cycles of 95 °C for 15 s and 60 °C for 30 s. Melt curve analysis was conducted to confirm product specificity. Gene expression was normalized to Gapdh using the 2^−ΔΔCt^ method. The sequence of primers for RT-PCR is listed in Supplementary Table S1.

### Behavioral tests

#### Rotarod test

The mice were tested on a rotarod with the velocity increasing from 4 rpm to 40 rpm within 5 min. The mice were pre-trained for 2 days for adaptation. Then the duration time on the rotarod before the mice fell off was recorded.

#### Von frey test

Double-blind behavioral tests were carried out. The von Frey test was used to assess the mechanical threshold. To identify the mice’s hindpaw reactions, we used progressive mechanical stimulation. Every stimulus was repeated five times. We counted the stimulus as positive if the mice withdrew and licked their hindpaw three or more times while resting. The mechanical threshold can then be defined.

#### Hargreaves test

Mice were habituated in plastic chambers, and radiant light was applied to one of their hindpaws. The radiant light was applied when the mice were resting quietly and was stopped immediately after the movement of the hindpaw. In the inflammatory pain model, 20 µL of CFA was intraplantarly injected into the hindpaw of each mouse, and we tested the noxious heat responses using Hargreaves test when applied radiant heat to the hindpaw with CFA injection.

#### Hotplate test

Mice were put on a hot plate at a temperature of 52 °C, and the cutoff time was 50 s.

#### Acute itch test

To observe the acute itch behaviors, the mice were acclimatized and followed by pruritic compounds injection. Drugs were injected intradermally into the nape of the neck or cheek, and scratching behavior was video-recorded for 30 min. A bout of scratching was defined as continuous scratch movements with hindpaws directed at the area around the injection site. The administered doses (per mouse) were: β-alanine (1000 μg/50 μL), chloroquine (CQ, 200 μg/50 μL), histamine (500 μg/50 μL), 5-hydroxytryptamine (5-HT, 10 μg/50 μL), and N-methyl leukotriene C4 (N-met LTC4, 0.75 μg/50 μL).

#### DNFB model

DNFB model was made as the following steps: 1. Mice were anesthetized with 2% isoflurane, removing fur on the abdomen area (1 cm × 1 cm). 2. Sensitization: DNFB is dissolved into acetone and olive oil mixture (4:1). For sensitization, 50 μL, 0.5% DNFB was administrated by intradermal injection, then 100 μL 0.5% DNFB was painted on the abdomen area. 3. Challenge: 5 days after sensitization, remove the fur on the nape of the neck, 50 μL 0.2% DNFB is painted on the nape of the neck. 4. Challenges were performed every other day, video the itch behavior 24 h after the challenge, and the itch behavior will be quantified (15 min after placing the mice into the behavioral cages).

#### AEW model

AEW model was made as following steps: 1. Mice were anesthetized with 2% isoflurane, removing fur on the nape of the neck (1 cm × 1 cm). 2. Acetone: ether (1:1) for 20 s, and water for 40 s, twice daily, 9:00 a.m. and 5:00 p.m. 3. The mice were treated with AEW for 10 consecutive days. 4. Itch behaviors were recorded with a video camera for 1 h.

#### CFA model

CFA-induced inflammation models were created according to earlier protocols (Liu X. J. et al., 2012). Briefly, a subcutaneous injection of 20 µL CFA was performed at the root of each toe and the center of both hindpaws.

#### SNI model

For the SNI test, the mice were anesthetized with isoflurane during the surgery. The tibial and common peroneal nerve branches were cut off, leaving the remaining sural nerve intact.

#### Formalin model

For the formalin test, formalin (0.5% in 1xPBS, 20 µL) was injected subcutaneously into the dorsal part of the left hindpaw, and the nociceptive responses of mice were video recorded for 1 h. The number of hindpaw flinches in each 5 min interval was counted.

### Immunohistochemistry

The antibody against Tmem45b was produced by GL Biochem company (For western blot, the antigenic peptide sequence is C-DHTYQSALLSGSDEE; For immunohistochemistry, the antigenic peptide sequence is C-RPEWDQKDMDN). The procedure as follows: Lumbar DRGs were fixed in 4% paraformaldehyde, cryoprotected in 20% sucrose, and sectioned at 10 μm thickness. Sections were blocked with 10% normal donkey serum in PBS containing 0.05% Triton X-100 for 1 h at room temperature, followed by incubation with primary antibodies overnight at 4 °C. After incubation, sections were washed three times with PBS (5 min each), then incubated with appropriate secondary antibodies for 1 h at room temperature. To test the antibody specification, the primary antibodies (1:2000) and antigenic peptide of Tmem45b (10^–5^ M) were mixed and rotated for 1h at RT and overnight at 4 °C. The images were collected with Leica SP8 confocal microscope. Antibodies used in this study: GM130 (BD, 610822), Th (Millipore, AB1542), IB4 (Vector, FL-1201-.5), Tuj1 (Starter, SDT-251-28), CGRP (Dia Sorin, 24112), NF200 (CST, 2836S), PDI (Santa Cruz, SC-20132), Calnexin (Abcam, ab112995), Serca1 (Proteintech, 22361-1-AP), mitochondria-tracer (Beyotime, C1048), TGN38 (Bio-Rad, AHP499G), GFAP (Millipore, MAB3402), IBA1 (Abcam, ab5076).

### RNAscope ISH

The probes and detection kit of RNAscope were purchased from ACD. For the fluorescent assay, the RNAscope Multiplex Fluorescent Detection Reagent v2 (Cat No. 323110) was applied. The experiments were performed according to the kit instructions. The sections from Lumbar DRGs were hybridized with these probes: Tmem45b (Cat No.420461), Mrgpra3 (Cat No. 548161-C2), Mrgprd (Cat No. 417921-C3), Nppb (Cat No. 425021-C3). For bright-field single RNA detection, RNAscope 2.5 HD Detection Kit (RED) (Cat# 322350) Mrgpra3 probe (Cat No. 548161) was used. The images were collected with Leica SP8 confocal microscope and SLIDEVIEW VS200 microscope, respectively.

### Calcium imaging

DRG neurons were incubated in the Ca^2+^ indicator Furo-4 (2 μM; Invitrogen) in ECS bath solution (in mM, 140 NaCl, 3 KCl, 2 MgCl_2_, 2 CaCl_2_ and 10 HEPES, pH 7.3) at 37 °C. Ca^2+^ free ECS solution (in mM, 140 NaCl, 3 KCl, 2 MgCl_2_, 1 EGTA and 10 HEPES, pH 7.3). High K solution (in mM, 140 NaCl, 40 KCl, 2 MgCl_2_, 2 CaCl_2_ and 10 HEPES, pH 7.3). Neurons that respond to 40 mM KCl are considered viable and are recorded. Fluorescence data were acquired on a PC running Metafluor software (Molecular Devices, Sunnyvale, CA, United States). Calcium-induced fluorescence was analyzed by ImageJ software and cell-based fluorescence was determined by defined regions of interest (ROI). The fluorescence value of the background is Fb, the fluorescence value of the first image was F0 and the fluorescence value over time was Ft. Data were calculated as F/F0 = (Ft–Fb)/F0. The drugs were administered using a gravity-driven syringe device. To stimulate DRG neurons, the following concentrations were used: CQ, 1 mM; Histamine, 1 mM; beta-alanine, 3 mM; 5-HT, 100 μM; Capsaicin, 300 μM; N-met LTC4, 100 nM; TG, 100 μM; Caffeine, 10 mM, Trypsin, 500 nM.

### Sucrose gradient density centrifugation

The DRGs were collected and homogenized in preparation buffer (320 mM sucrose, 4 mM HEPES, pH 7.4) using a Pestle-glass homogenizer at 4 °C. The homogenate was centrifuged for 10 min at 1,000 g. The pellet was discarded, while the supernatant was collected and centrifuged for 15 min at 9,200 g. The pellet was resuspended in sucrose buffer (200 mM sucrose, 0.1 mM MgCl_2_, 0.5 mM EGTA, 10 mM HEPES, pH 7.4). The suspension was applied to a 10%–60% (w/v) continuous sucrose gradient prepared in 4 mM HEPES (pH 7.4), and the gradient was centrifuged at 150,000 g for 3 h. The protease inhibitors aprotinin, leupeptin, pepstatin, and PMSF were added to all solutions. The samples were processed for SDS-PAGE.

### Cell culture and transfection

COS7 cells were cultured in DMEM with 10% fetal bovine serum. The cells were transiently transfected with plasmids using Lipofectamine 2000 reagent (Invitrogen) and were used for the following various experiments 24–48 h after transfection. The Tmem45b plasmid was purchased from origene company (Cat. No MG203741).

### Western blot analysis

Tissue samples were homogenized in RIPA buffer (150 mM NaCl, 30 mM HEPES, 10 mM NaF, 1% Triton X-100, and 0.01% SDS) with protease inhibitors (1 mM PMSF, 10 mg/mL aprotinin, 1 mg/mL pepstatin, and 1 mg/mL leupeptin). The samples were denatured and loaded for SDS-PAGE and then transferred to the nitrocellulose membrane. Primary antibody was applied overnight at 4 °C, and secondary antibodies were applied for 1 h at room temperature. The specific protein bands were visualized with chemiluminescence. The primary antibodies include that against GAPDH (ab8245, Abcam), the western blot was repeated with 3 independent experiments.

### Identification of cell clusters and visualization of gene expression levels

Data were filtered, processed, and analyzed using the Seurat (V4.3.0.1) package in R (V4.3.1). Filtering of initial data involved selecting cells with >2000 features and <20 mitochondrial genes, then the clean data were clustered and annotated. DRG neuron types were categorized according to the classification mentioned previously, including 8 types and 16 subtypes based on 10x Genomics technology. Clusters were annotated based on specifically expressed genes, and the annotated cells were clustered again while removing the cell expressing another type-specific gene, which was doublets. Finally, the expression levels of the genes in the different neuron types were presented using the R package ggplot2 (V3.4.3) and the VlnPlot function within Seurat (V4.4.0).

### Bulk RNA-seq analysis

The extracted RNA samples were subjected to high-throughput sequencing on the DNBseq platform with a read length of PE150. The raw sequencing data underwent quality control, during which low-quality sequences and adapter contaminants were removed using fastp (V0.23.2). Cleaned reads were then aligned to the reference genome using bwa-mem2 (V2.2.1), and sorted using samtools (V1.14) to generate the count matrix. Differential expression gene analysis was performed using DESeq2 (V1.40.2), where the count data were first normalized, and a negative binomial distribution model was employed to calculate p-values and adjusted p-values (padj) for each gene. A significance threshold of padj <0.1 and |log2FC| > log2 (1.5) was set to identify upregulated and downregulated genes.

### Venn diagram

The sequencing and raw data processing were performed using the methods mentioned earlier. Differentially expressed gene analysis was conducted using DESeq2 (V1.40.2), with a significance threshold set at padj < 0.1 and |log2FC| > log2 (1.5) to filter for DEGs. The Venn diagram was created using the R package VennDiagram (V1.7.3), with DEGs in the WT group marked in red, DEGs in the CKO group marked in blue, and DEGs present in both groups marked in purple.

### Gene ontology enrichment analysis

After filtering for DEGs, gene ontology enrichment analysis was performed using the function compareCluster from the R package clusterProfiler (V4.8.3) for multiple gene sets, displaying the top 10 enriched pathways. The adjusted p-value (p.adjust) was chosen as the significance threshold, and GeneRatio was used to represent the relative abundance of genes in the pathways, with visualization done using ggplot2 (V3.4.4).

### DRG culture and siRNA knockdown

DRGs were isolated from the C57BL/6J mice and dissociated as previously described. Briefly, total DRGs were dissected and digested in oxygenated DMEM containing 1 mg/mL collagenase (Sigma, C9891), 0.4 mg/mL trypsin (Sigma, T8003), and 0.1 mg/mL DNase I (Sigma, DN25) at 37 °C for 30 min. The resulting cell clusters were gently triturated using flame-polished glass pipettes and filtered through 70 μm nylon cell strainers (Falcon, No. 352350). Myelin debris was removed using 10% percoll (Sigma, P1644). All centrifugations steps were performed at 200 × g, 3 min. Then, the cells were planted in the 12-well plates with poly-D-lysine-coating. The cells were cultured in an F12 medium with 10% FBS. The siRNAs were transfected into DRG neurons by lipofectamine RNAi MAX (Invitrogen, Cat No. 13778). The concentration of siRNA (GenePharma) added to the cultured neurons was 100 nM. The sequence of scramble siRNA was sense 5-UUCUCCG-AACGUGUCACGUTT-3, antisense 5-ACGUGACACGUUCGGAGAATT-3. The sequence of Tmem45b was 5-CCA-UCAACUACUCCCUUGUTT-3, antisense 5-ACAAGGGAGUAGUUGAUGGTT-3.

### *In vivo* siRNA injection

Mice were habituated for 2 days prior to the start of behavioral experiments. Subsequently, the animals received intrathecal injections of 4 μg/10 μL siTmem45b or scramble siRNA once daily for two consecutive days. The siRNAs (GenePharma) were modified with 2′-O-methylation (2′-OME) and 5′-cholesterol (5′-Chol) to enhance stability and uptake. The sequences used were identical to those applied in the transfection experiments. For *in vivo* delivery, the siRNAs were complexed with *in vivo* jetPEI (Polyplus, Cat. No. 201-50G), and the procedure was performed according to the manufacturer’s instructions. After the behavioral experiments, cervical DRGs were collected for qPCR analysis to assess knockdown efficiency.

### Toluidine blue staining and hematoxylin and eosin (H&E) staining

The skin were fixed in 4% paraformaldehyde for 10 min. After rinsing in PBS, the skin were stained with the toluidine blue staining kit according the instruction (Sangon biotech) and H&E staining (Beyotime), respectively. Then the skin were rinsed with distilled water, air-dried, and mounted. Images were collected with SLIDEVIEW VS200 microscope.

### Statistical analysis

All statistical analyses were performed using GraphPad Prism 9.0. The data were presented as means ± SEM. Student’s t-test performed statistical analysis for two cohorts. Two-way ANOVA performed comparisons between multiple cohorts with Bonferroni’s *post hoc* test. P < 0.05 was considered statistically significant.

## Results

### Tmem45b is primarily expressed in itch-sensing neurons

**FIGURE 1:**
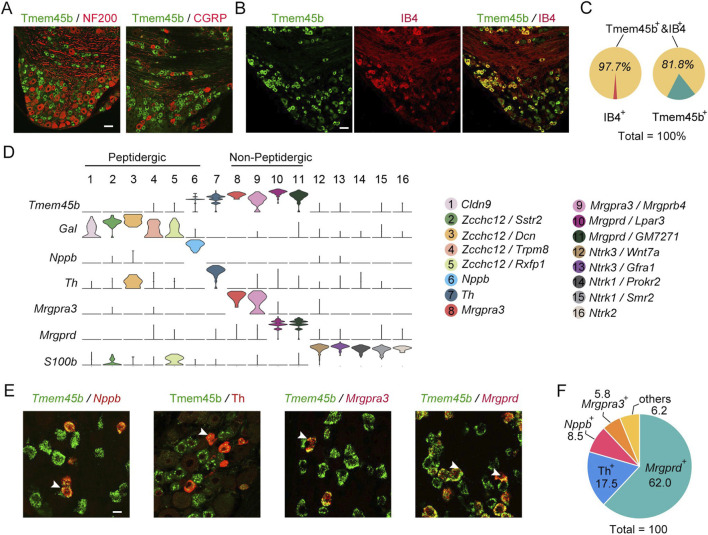
*Tmem45b* is primarily expressed in itch-sensing neurons. **(A)** Immunostaining results show the expression of Tmem45b (green), CGRP (red), and NF200 (red). **(B)** Immunostaining results show the co-expression of Tmem45b (green) and IB4 (red). **(C)** Statistical analysis shows the proportion of Tmem45b^+^IB4^+^. **(D)** scRNA-seq (10x Genomics) analysis identified 16 subtypes of DRG neurons. Gene annotations for these 16 neuron types are shown in the right panel. Tmem45b is predominantly enriched in neurons positive for *Mrgprd, Mrgpra3, Nppb*, and *Th*. **(E)** RNAscope *in situ* hybridization shows co-localization among Tmem45b (green), *Mrgprd* (red)*, Mrgpra3* (red), and *Nppb* (red). Immunostaining results show that Tmem45b (green) is co-expressed with Th (red). Arrows indicate co-expressing cells. Scale bar, 20 μm. **(F)** Proportions of *Mrgprd* ^+^, *Mrgpra3* ^+^, *Nppb* ^+^, and Th^+^ neurons among Tmem45b^+^ DRG neurons. DRG was obtained from at least 3 mice.

To determine the tissue-specific expression of Tmem45b, we generated a rabbit polyclonal antibody (see Methods). The specificity of the antibody was confirmed through antigen absorption experiments using both immunohistochemistry and western blot analysis (Supplementary Figure S1A,B). To assess the expression pattern of Tmem45b in DRG neurons, we performed immunohistochemistry to examine its co-localization with IB4, NF200 (label large DRG neurons), and CGRP. The results showed that Tmem45b was rarely co-expressed with NF200 and CGRP (Figure 1A). In comparison, Tmem45b was expressed in 97.7% of IB4-positive sensory neurons, while IB4 was present in 81.8% of Tmem45b^+^ neurons (Figures 1B,C). According to scRNA-seq analysis, we classified DRG neurons into 16 subtypes, including Claudin-9 (Cldn9)-, zinc-finger CCHC domain-containing 12 (Zcchc12)-, Nppb-, Th-, Mrgpra3-, Mrgprb4-, Mrgprd-, neurotrophic receptor tyrosine kinase (Ntrk3)-, Ntrk1-, and Ntrk2-positive neurons (Figure 1D) (Li et al., 2016). Mrgprd^+^ and Mrgpra3^+^ neurons represented nonpeptidergic neurons that were labelled by IB4 (Li et al., 2016). Reanalysis on scRNA-seq data revealed that Tmem45b was expressed not only in Mrgpra3^+^ neurons and Mrgprd^+^ neurons, but also in Nppb^+^ and Th^+^ neurons (Figure 1D). Further results of RNAscope *in situ* hybridization (ISH) and immunohistochemistry showed that Tmem45b^+^ neurons consisted of approximately 62% Mrgprd^+^, 8.5% Nppb^+^, 5.8% Mrgpra3^+^, and 17.5% Th^+^ neurons (Figures 1E,F). Taken together, these findings showed that Tmem45b was predominantly expressed in itch-sensing neurons within the DRG.

### Conditional deletion of Tmem45b alleviates the mechanical inflammatory pain

**FIGURE 2:**
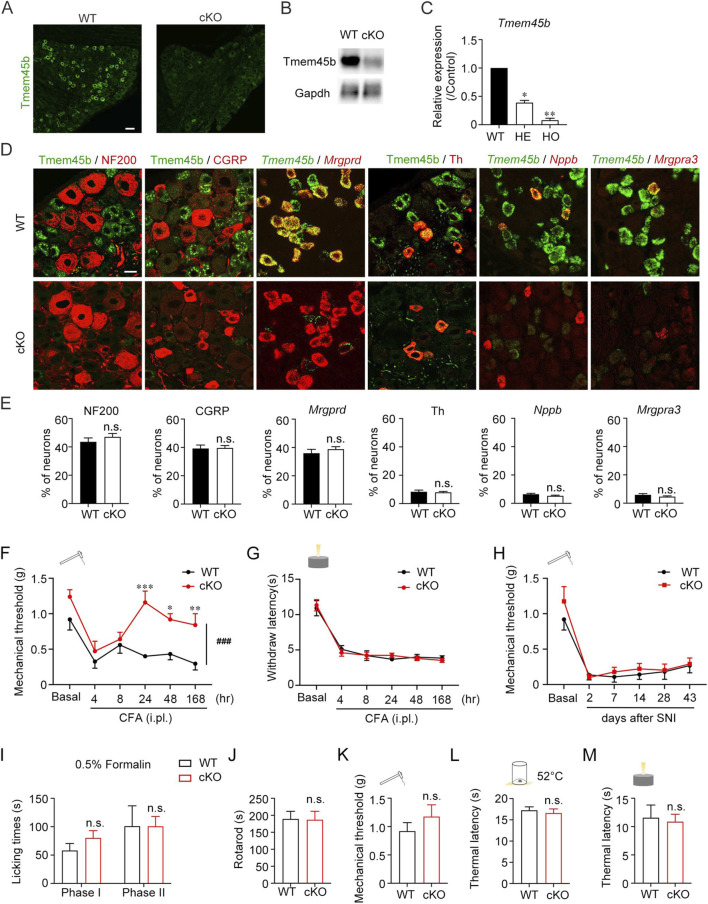
Conditional deletion of Tmem45b alleviates the inflammation-induced mechanical pain. **(A)** Immunohistochemistry and **(B)** western blot results show the expression of Tmem45b. Scale bar, 50 μm. **(C)** qPCR results show the expression of Tmem45b mRNA. **(D)** RNAscope ISH results show the expression of Tmem45b, Mrgprd, Nppb, and Mrgpra3. Immunohistochemistry results show the expression of NF200, CGRP, and Th. **(E)** Statistical analysis. Two-tailed unpaired Student’s t test. **(F–I)** Effects of Tmem45b cKO on pain hypersensitivity in different pain models: **(F)** CFA-induced inflammation pain (WT vs. cKO, n = 5 vs. 5). **(G)** CFA-induced inflammation thermal pain (WT vs. cKO, n = 5 vs. 12), **(H)** SNI-induced neuropathic pain (WT vs. cKO, n = 5 vs. 8). Two-way ANOVA test. **(I)** Formalin-induced chemical pain (WT vs. cKO, n = 5 vs. 5), Two-tailed unpaired Student’s t test. **(J)** Rotarod test showed the motor activity. **(K–M)** Von frey, hot plate and hargreaves showed the mechanical threshold and noxious thermal sensation of WT and cKO mice (WT vs. cKO, n = 5 vs. 5). Two-tailed unpaired Student’s t test. Data are presented as Mean ± S.E.M. *P < 0.05, **P < 0.01, ***P < 0.001, ^###^ *P* < 0.001.

In previous studies, a systemic Tmem45b knockout mouse was generated by deleting a 3.1 kb genomic fragment of Tmem45b using the CRISPR/Cas9 system (Tanioku et al., 2022). Since systemic knockout models may induce compensatory effects, we opted to conduct behavior studies using cKO mice. Mrgprd^+^ neurons comprised the largest subset of Tmem45b^+^ neurons, and represent a major population of nonpeptidergic nociceptors involved in regulating itch and mechanical allodynia. To investigate the role of Tmem45b in these neurons, we bred Mrgprd-Cre::Tmem45b^flox/flox^ mice, allowing for Tmem45b cko in Mrgprd^+^ neurons. Tmem45b^flox/flox^ mice were used as the WT group. Both immunohistochemistry and western blot analysis showed a significant reduction of Tmem45b protein levels in DRG from cKO mice (Figures 2A,B). Immunohistochemistry analysis revealed that Tmem45b^+^ neurons accounted for 37.5% of DRG neurons in WT mice, 39.6% in heterozygotes, and only 7.1% in homozygotes (Supplementary Figure S2A). Consistently, qPCR analysis confirmed a 61% reduction in Tmem45b mRNA level in heterozygotes and a 90% reduction in homozygotes (Figure 2C), indicating efficient knockout in homozygotes, which were used for subsequent analyses. To determine whether Tmem45b knockout affects the distribution of peripheral nerve terminals, we examined the expression of Tuj1 and CGRP in both hairy and glabrous skin of Tmem45b cKO mice. Quantitative analysis showed no significant changes in total free nerve endings and peptidergic free nerve endings, respectively (Supplementary Figures S1C–F). Next, we analyzed the central projections of both non-peptidergic and peptidergic DRG neurons in the dorsal horn of the cervical and lumbar spinal cord of Tmem45b cKO mice. Statistical analysis showed no significant changes in the distributions of either IB4 or CGRP in spinal dorsal horn (Supplementary Figures S2G–L).

To assess whether Tmem45b knockout affect DRG neuron development, we examined the neuron subtype distribution. RNAscope ISH and immunohistochemistry revealed no significant changes in the percentages of NF200^+^, CGRP^+^, *Mrgprd* ^+^, Th^+^, *Nppb* ^+^, and *Mrgpra3* ^+^ neurons between WT and cKO mice (Figures 2D,E). Notably, we found that Tmem45b expression was abolished not only in Mrgprd^+^ neurons but also in Mrgpra3^+^ and Nppb^+^ neurons (Figure 2D). This finding indicated that Tmem45b expression was abolished in the itch-sensing neurons. In contrast, Tmem45b expression was retained in Th^+^ neurons (Figure 2D). qPCR results indicated that the mRNA level of neuronal marker genes such as *Gal*, *Mrgprd*, *Mrgpra3*, *Th*, *Nppb*, *S100b*, and *Calca* were not altered in Tmem45b cKO mice (Supplementary Figure S2C). We employed RNAscope ISH with Fast Red dye under bright-field conditions, a highly sensitive method to detect single RNA transcript, to carefully evaluate Mrgpra3 expression. The results showed that the signal intensity of individual Mrgpra3 RNA molecules at the single-cell level was elevated, and the proportion of Mrgpra3^+^ neurons was not changed in Tmem45b cKO mice (Supplementary Figures S2N–O). Furthermore, dual-fluorescence RNAscope ISH results demonstrated that Tmem45b knockout did not lead to an upregulation of Mrgpra3 in Mrgprd^+^ neurons (Supplementary Figure S2P,Q). TRP channels are critical for sensory transmission. TRPA1 was required for the nonhistaminergic itch (Cavanaugh et al., 2009). TRPV1 is involved in noxious heat perception, and TRPM8 is essential for cold sensation (Voets et al., 2004). qPCR results showed that the mRNA levels of *Trpa1*, *Trpv1*, and *Trpm8* were not significantly changed in Tmem45b cKO mice (Supplementary Figures S2J–L). The receptors of growth factors including TrkA, TrkB and TrkC are important for the development and function of DRG neurons. qPCR results showed that *Ntrk1*, *Ntrk2*, and *Ntrk3* expression were not altered in Tmem45b cKO mice (Supplementary Figure S1M).

Next, we assessed the impact of Tmem45b loss in itch-sensing neurons on nociceptive responses. Both Complete Freund’s adjuvant (CFA)-induced inflammation pain model and spared nerve injury-induced neuropathic pain model decreased the withdrawal threshold to mechanical stimuli (mechanical allodynia). Tmem45b cKO mice exhibited a markedly increased mechanical threshold to von Frey filaments (mechanical test) from 24 to 168 h after intraplantar CFA injection (Figure 2F). However, the loss of Tmem45b did not affect the mechanical allodynia within the first 8 h (Figure 2F). These results were different from previous findings (Tanioku et al., 2022). CFA injection could also reduce the paw withdrawal latency to noxious heat stimuli (thermal hyperalgesia). The withdrawal latency in Hargreaves test (thermal test) was not different between Tmem45b cKO and WT mice (Figure 2G). Bulk RNA-seq was performed on the lumbar DRGs from mice 7 days post-CFA injection. We compared naïve and CFA-treated WT mice, as well as naïve and CFA-treated cKO mice (Supplementary Figure S2R,S). Gene ontology enrichment analysis showed that both WT and cKO mice with inflammation shared the genes related to extracellular matrix components, such as *Mmp9* and *Aebp1*, which were involved in tissue repair and remodeling processes (Blackburn et al., 2018; LeBert et al., 2015). However, we observed *Mmp2* and *Ccr2* were enriched in CFA-treated cKO groups (Supplementary Figure S2S), which were related to leukocyte chemotaxis and migration (Raghu et al., 2017; Song et al., 2015). The migration of neutrophils and macrophages could influence the intensity and duration of inflammation through DRG neurons (Lu et al., 2024).

Besides, Tmem45b cKO mice showed long-lasting mechanical allodynia following spared nerve injury from 2 to 43 days (Figure 2H). These findings demonstrated that Tmem45b was important for the maintenance of inflammation-induced mechanical allodynia but dispensable for thermal hyperalgesia, as well as neuropathic pain. We next asked whether Tmem45b deficiency contributed to pain sensitivity in formalin-induced chemical pain model. The licking time during either phase I (acute pain) or phase II (prolonged pain) was not significantly change between cKO and WT mice (Figure 2I). Besides, the rotarod test showed that Tmem45b cKO mice had no defects in motor ability (Figure 2J). Next, we examined the mechanical threshold and paw withdrawal latency to noxious heat stimuli under normal conditions. No differences were observed (Figures 2K–M). This suggested that Tmem45b was not involved in mechanical sensation or noxious heat sensation under normal conditions, consistent with a previous study (Tanioku et al., 2022). Taken together, Tmem45b was selectively involved in the maintenance of inflammation mechanical allodynia.

### Tmem45b deficiency modulates β-alanine- and CQ-induced itch

**FIGURE 3:**
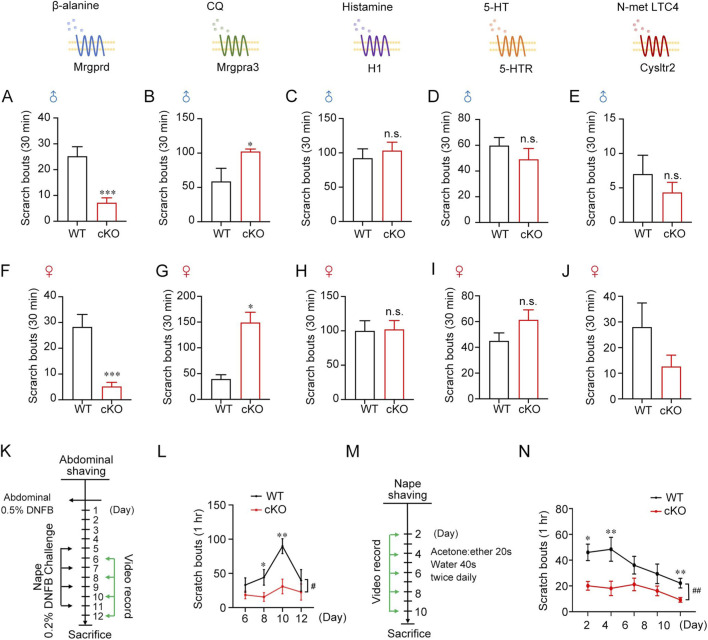
Tmem45b deficiency inhibits β-alanine-itch while enhanced CQ-induced itch. **(A,B)** Behavioral analysis of the itch evoked by β-alanine (1000 μg/50 μL) (WT vs. cKO, n = 10 vs. 12), and CQ (200 μg/50 μL), (WT vs. cKO, n = 5 vs. 6). **(C–E)** Behavioral analysis of the itch evoked by histamine (500 μg/50 μL), (WT vs. cKO, n = 5 vs. 8), 5-HT (10 μg/50 μL), (WT vs. cKO, n = 5 vs. 8), and N-met LTC4 (0.75 μg/50 μL), (WT vs. cKO, n = 9 vs. 11). **(F,G)** Behavioral responses to intradermal injection of β-alanine (WT vs. cKO, n = 10 vs. 12) and CQ (WT vs. cKO, n = 5 vs. 22). **(H–J)** Behavioral responses to intradermal injection of 5-HT (WT vs. cKO, n = 5 vs. 10), Histamine (WT vs. cKO, n = 5 vs. 10), and N-met LTC4 (WT vs. cKO, n = 5 vs. 10). Two-tailed unpaired Student’s t test. **(K)** Schematic of DNFB model. **(L)** Quantification of scratching bouts in DNFB model (WT vs. cKO, n = 5 vs. 10). **(M)** Schematic of AEW model. **(N)** Quantification of scratching bouts in AEW model (WT vs. cKO, n = 16 vs. 14). Two-way ANOVA test. Data are presented as Mean ± S.E.M. *P < 0.05, **P < 0.01, ***P < 0.001; ^#^ *P* < 0.05, ^##^ *P* < 0.01.

Next, we investigated the impact of Tmem45b deficiency on itch sensation. Itch is broadly characterized as either histaminergic or nonhistaminergic (Yosipovitch et al., 2018). The responses of Tmem45b cKO and WT mice to intradermal injections of various pruritogens were assessed. In addition to histamine, nonhistaminergic agents were used, such as β-alanine, CQ, serotonin (5-HT), and N-met LTC4. The β-alanine-evoked scratching bouts were significantly reduced in Tmem45b cKO mice (Figure 3A). In contrast, CQ-induced scratching bouts were significantly increased (Figure 3B). β-alanine and CQ exhibited opposite effects in Tmem45b cKO mice. To ensure the reliability of our conclusions, we confirmed these findings in female mice. Behavioral phenotypes of female mice were consistent with those of male mice (Figures 3F,G). Besides, we adopted the cheek model to assess pruritic behavior. Tmem45b cKO mice similarly exhibited reduced scratching in response to β-alanine, while enhanced scratching to CQ (Supplementary Figures S3A,B). Additionally, no significant differences were observed in the responses to 5-HT or histamine (Figures 3C,D,H,I). N-met LTC4 can bind to Cysltr2 and activate Nppb^+^ DRG neurons to induce itch (Solinski et al., 2019). Tmem45b cKO mice showed comparable scratching behavior compared to WT (Figures 3E,J). Taken together, Tmem45b deficiency selectively inhibited β-alanine-evoked itch while enhancing CQ-evoked itch.

Moreover, the role of Tmem45b in chronic itch was investigated. Two distinct chronic itch models were applied. Atopic dermatitis itch was induced by Hapten 1-Fluoro-2,4-dinitrobenzene (DNFB), and dry skin itch was caused by the acetone/ether/water (AEW) treatment (Miyamoto et al., 2002; Wang Z. et al., 2021). In the DNFB model, the number of scratching bouts was significantly reduced in Tmem45b cKO male (Figures 3K,L) and female mice (Supplementary Figure S4A,B). Notably, female mice showed significantly more frequent scratching behavior than males in the DNFB model (Supplementary Figure S4B) since previous studies demonstrated that females exhibit stronger immune responses than males in immune-related diseases (Leyden and Kligman, 1977). This gender-specific difference persisted without attenuation on day 10 (Supplementary Figure S4B). To explore the therapeutic potential of Tmem45b in atopic dermatitis-like itch, we conducted DNFB sensitization in C57BL/6 mice and administered intrathecal injections of either scramble siRNA or Tmem45b siRNA 2 days prior to challenge (Supplementary Figure S4C). Intrathecal injection of Tmem45b siRNA significantly reduced Tmem45b mRNA levels, as confirmed by qPCR. Behavioral analysis revealed that the scratching behavior in the Tmem45b siRNA-treated group was significantly lower than that in the control group on days 1, 3, and 5 after DNFB challenge (Supplementary Figure S4D,E). Subsequently, we examined Tmem45b expression in DRGs of DNFB-treated mice at days 6, 8, 10, and 12. The results showed that DNFB-induced chronic itch had no effect on Tmem45b expression in DRGs (Supplementary Figure S4F). This finding suggests that Tmem45b is necessary for atopic dermatitis-like chronic itch behavior. Immune cells, especially mast cells, are key players in the development of chronic itch. Toluidine blue staining results showed that the number of mast cell in the basal region of the skin was significantly lower in Tmem45b cKO mice on day 10 of DNFB model (Supplementary Figure S4H,I). A Similar loss of scratching behavior was observed for Tmem45b cKO mice following the AEW treatment (Figures 3M,N), indicating that Tmem45b deficiency inhibits chronic itch. Next, we analyzed H&E staining of WT and Tmem45b cKO mouse skin on day 0, day 2, day 4 and day 10 following AEW treatment. The results showed that on AEW day 4 and day 10, the epidermal thickness in Tmem45b cKO mice was significantly lower than that in the WT group (Supplementary Figure S4J–K). Chronic itch is associated with the activation of spinal microglial cells, and inhibition of microglial activation has been shown to reduce scratching behavior (Du et al., 2019; Zhang et al., 2015). Moreover, reactive astrocytes and lipocalin-2 contribute to chronic itch by sensitizing the GRP/GRPR pathway (Koga et al., 2020; Shiratori-Hayashi et al., 2015). Finally, to assess glial activation, we examined the expression of IBA1 (label microglia) and GFAP (label astrocytes) in the cervical enlargement of the spinal cord. Immunohistochemical analysis revealed a significant reduction in both the total signal intensity and the area ratio of astrocytes in Tmem45b cKO mice (Supplementary Figure S5A–C). A similar reduction was observed for microglial cells (Supplementary Figure S5D). In line with these, qPCR results showed the considerable downregulation of astrocyte markers (*Aldh1l1* and *Gfap*) and microglial markers (*Csf1r* and *Cx3cr1*) in the cervical spinal cord of cKO mice (Supplementary Figures S5G–J). Taken together, these findings demonstrated that Tmem45b deficiency reduced chronic itch and inhibited glial activation in the spinal cord.

### Tmem45b deficiency impairs calcium responses to β-alanine and AITC

**FIGURE 4:**
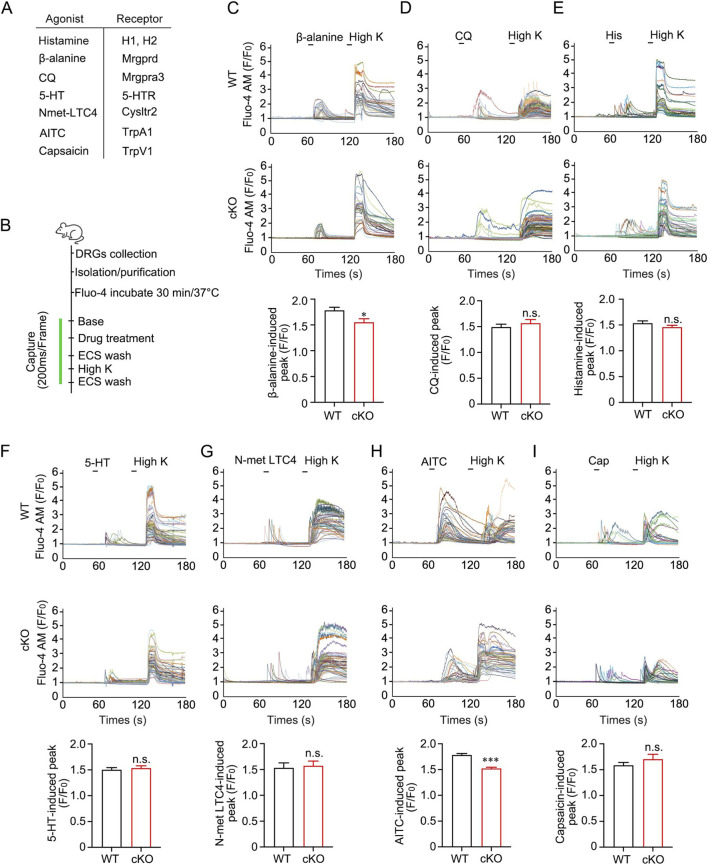
Tmem45b deficiency inhibits the calcium response to β-alanine and AITC. **(A)** The table shows the agonists and their corresponding receptors. **(B)** Schematic of calcium imaging in dissociated DRG neurons. **(C)** Representative calcium imaging results and statistical analysis of responses to β-alanine (3 mM), (WT vs. cKO, n = 75 vs. 27). **(D)** Representative calcium imaging results and statistical analysis of responses to CQ (1 mM), (WT vs. cKO, n = 40 vs. 32). **(E)** Representative calcium imaging results and statistical analysis of responses to histamine (1 mM), (WT vs. cKO, n = 110 vs. 82). **(F)** Representative calcium imaging results and statistical analysis of responses to 5-HT (100 μM), (WT vs. cKO, n = 143 vs. 161). **(G)** Representative calcium imaging results and statistical analysis of responses to N-met LTC4 (100 nM), (WT vs. cKO, n = 24 vs. 43). **(H)** Representative calcium imaging results and statistical analysis of responses to AITC (100 μM), (WT vs. cKO, n = 346 vs. 248). **(I)** Representative calcium imaging results and statistical analysis of responses to capsaicin (300 nM), (WT vs. cKO, n = 84 vs. 51). F represents the calcium fluorescence, and F_0_ represents the fluorescence of the first image. Two-tailed unpaired Student’s t test. DRG was obtained from at least 3 mice. Data are expressed as the Mean ± S.E.M. *P < 0.05, ***P < 0.001.

To investigate the mechanisms underlying the effect of Tmem45b on itch behavior, we conducted *in vitro* calcium imaging on dissociated DRG to assess their responses to β-alanine, CQ, histamine, 5-HT, and N-met LTC4 (Figures 4A,B). Neuronal calcium responses to different drugs were recorded. The level of peak calcium signal induced by β-alanine (1 mM, WT vs. cKO, n = 75 vs. 27) was significantly lower in cKO DRG neurons (Figure 4C). This result was consistent with the reduced β-alanine-induced itch response observed on Tmem45b deficiency mice (Figure 3A). Next, we asked whether Tmem45b deficiency could enhance the calcium response to CQ. To our surprise, we did not observe any significant differences in calcium activity in response to CQ (1 mM, WT vs. cKO, n = 40 vs. 32) between cKO and WT neurons (Figure 4D). This contrasted with our behavioral observations, which showed Tmem45b deficiency enhanced CQ-evoked itch (Figure 3B). In contrast, calcium response to histamine (1 mM, WT vs. cKO, n = 110 vs. 82), 5-HT (100 μM, WT vs. cKO, n = 143 vs. 161) and N-met LTC4 (100 nM, WT vs. cKO, n = 24 vs. 43) did not differ between cKO and WT DRG neurons (Figures 4E–G). The calcium imaging results were consistent with previous behavioral observations, which showed no significant changes in itch responses to these pruritogens (Figures 3C–E).

TRPA1 and TRPV1 are essential for itch mediated by Mrgpr signaling, including Mrgpra3 and Mrgprd (Han et al., 2013; Wilson et al., 2011). We next examined the TRPA1 and TRPV1 function in dissociated DRG neurons with the agonist AITC (100 μM, WT vs. cKO, n = 346 vs. 248) and capsaicin (300 nM, WT vs. cKO, n = 84 vs. 51), respectively. Calcium imaging analysis showed that the peak calcium response to AITC was markedly reduced in Tmem45b cKO neurons (Figure 4H). This finding was consistent with the reduction in both β-alanine-induced calcium flux and itch behavior. However, Tmem45b cKO neurons showed no significant change in the peak calcium responses following capsaicin treatment (Figure 4I). Besides, TRPV1 is critical for the histamine-dependent itch (Shim et al., 2007). This aligns with our prior finding that Tmem45b cKO mice showed no significant alteration in histamine-induced acute itch behavior. Collectively, these results indicated that the lack of Tmem45b impaired TRPA1 function rather than TRPV1.

The discrepancy between the calcium responses and behavioral results following CQ stimulation led us to hypothesize that, beyond classical TRP channels, additional molecular components may contribute to the activation of *Mrgpra3* ^*+*^ neurons by CQ. Through scRNA-seq analysis, we identified the different expression pattern of *Trpa1* and *Trpv1* in Mrgprd^+^ and Mrgpra3^+^ neurons. *Trpv1* showed higher expression in Mrgpra3^+^ neurons, whereas *Trpa1* showed lower expression in Mrgpra3^+^ neurons than in Mrgprd^+^ neurons (Supplementary Figure S6A). In addition to *Trpa1* and *Trpv1*, other genes such as *Prkcq*, *Jph2*, *Kcnmb1*, and *Adora2b* also exhibit differential expression. These genes play important roles in regulating calcium responses and neuronal excitability (Castillo et al., 2015; Hu et al., 2016; Takeshima et al., 2015). Besides, we also compared the differences in KEGG pathways among itch-sensing neuronal subtypes: Nppb, Mrgpra3, and Mrgprd (Supplementary Figure S6B). Leukocyte transendothelial migration/cytokine receptor/neuroactive ligand receptor signaling pathway were enriched in Nppb^+^ neurons. Chemokine/VEGF/Toll-like receptor signaling pathway were enriched in Mrgpra3^+^ neurons. These pathways play important roles in itch transmission (Du et al., 2022; Yang et al., 2020; Zhang et al., 2021). PPAR/NOD-like receptor/Wnt signaling pathway were enriched in Mrgprd^+^ neurons (Gabrielli et al., 2024; Madahar et al., 2024; Tang et al., 2024). These pathways were essential for both pain and itch sensation. These findings also highlight the molecular complexity underlying itch signaling mediated by Mrgpra3^+^ and Mrgprd^+^ neurons.

### Tmem45b deficiency impairs ER calcium regulatory capacity

**FIGURE 5:**
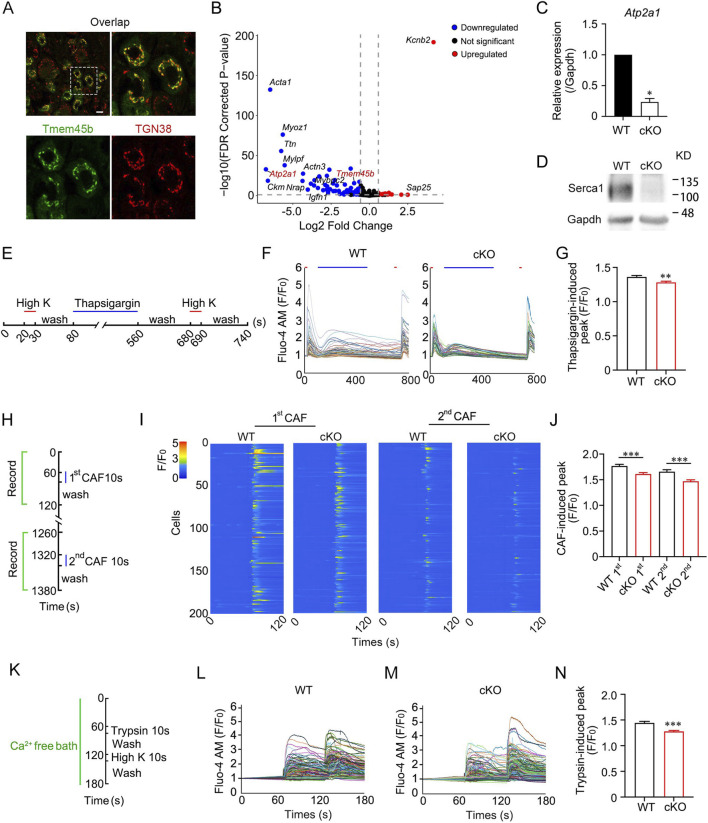
Tmem45b is essential for the calcium regulation of ER. **(A)** Representative immunostaining images show the colocalization between Tmem45b (green) and TGN38 (red) in the DRG tissue sections. Scale bar, 20 μm. **(B)** The volcano plot illustrates the significant differences between WT and CKO mice (WT vs. cKO, n = 3 vs. 4), assessed based on the fold change (cKO/WT) and adjusted p-value (padj). Blue dots represent genes that are significantly downregulated (fold change <0.67; padj <0.1), red dots indicate significantly upregulated genes (fold change >1.5; padj <0.1). Genes represented by black dots show no significant differences between the groups. **(C)** qPCR analysis. (WT vs. cKO, n = 4 vs. 4). Columns represent the mean expression level of Atp2a1 mRNA normalized to *Gapdh*. **(D)** Western blot analysis. Experiments are repeated three times. **(E)** Schematic of calcium imaging in dissociated DRG neurons response to thapsigargin. **(F)** Representative calcium imaging response to thapsigargin (100 μM). **(G)** Statistical results. (WT vs. cKO, n = 90 vs. 72). **(H)** Schematic of calcium imaging in dissociated DRG neurons in response to caffeine (10 mM). **(I)** The heatmap shows the calcium activity of DRG neurons in response to two rounds of caffeine stimulations. Neuron numbers are shown on the left of the image. **(J)** Statistical results. **(K)** Schematic of calcium imaging in dissociated DRG neurons response to Trypsin (500 nM). **(L, M)** Representative calcium response to Trypsin. **(N)** Statistical results. (WT vs. cKO, n = 227 vs. 217). Two-tailed unpaired Student’s *t* test. DRG was obtained from at least three mice. Data are expressed as the Mean ± S.E.M. *P < 0.05, **P < 0.01, ***P < 0.001.

To clarify the molecular mechanism underlying that Tmem45b affected neuronal calcium activity, we first confirmed the subcellular location of Tmem45b. Double immunohistochemistry staining in DRG tissues revealed that Tmem45b colocalized with TGN38, a trans-Golgi membrane marker (Figure 5A), which was consistent with a previous study (Tanioku et al., 2022). Additionally, sucrose density gradient ultracentrifugation showed that Tmem45b was presented in fractions containing GM130 (a cis Golgi marker) and PDI (an ER marker) (Supplementary Figure S7A). To further confirm the subcellular location of Tmem45b, we overexpressed the pCMV-Tmem45b-GFP plasmids in COS7 cells (Supplementary Figure S7B). However, GFP did not colocalize with GM130, Calnexin (an ER marker), or the mitochondrial tracer, confirming its expression on the trans-Golgi membrane (Supplementary Figures S7C–E).

To explore the molecular candidates mediating the change of calcium activity, we analyzed the bulk RNA-seq data of lumbar DRG (L4/5/6) from cKO and WT mice. Genes related to ER calcium regulation were identified (Figure 5B). We found that ATPase sarcoplasmic/endoplasmic reticulum Ca^2+^ transporting 1 (Serca1, encoded by *Atp2a1*), actinin alpha 3 (*Actn3*), creatine kinase muscle (*Ckm*), nebulin-related anchoring protein (*Nrap*), and titin (*Ttn*) were significantly downregulated. Notably, Serca is critical for sensory neurons sequester cytosolic Ca^2+^ and thereby maintain intracellular Ca^2+^ homeostasis (Wuytack et al., 2002). Serca family has three members, including Serca1 (*Atp2a1*), Serca2 (*Atp2a2*), and Serca3 (*Atp2a3*) (Periasamy and Kalyanasundaram, 2007). In Tmem45b cKO DRG, *Atp2a1* expression was significantly reduced, while *Atp2a2* and *Atp2a3* expressions remained unchanged (Supplementary Figure S8B). qPCR and western blot analysis confirmed that both Serca1 mRNA and protein levels were significantly reduced in Tmem45b cKO DRG (Figures 5C,D). To assess whether the reduction in Serca1 affected its function, we measured ER calcium release in dissociated DRG neurons. ER calcium stores could be depleted in the presence of thapsigargin, and neuron exposure to thapsigargin could cause bulk increases in cytosolic Ca^2+^ levels (Duncan et al., 2013; Sehgal et al., 2017). To exclude the effects of calcium channels on the cell membrane, we used Ca^2+^-free extracellular solution (ECS) (Figure 5E). The level of peak calcium signal induced by thapsigargin was markedly lower in cKO neurons (Figures 5F,G). This finding demonstrated that internal calcium flux released from the ER was significantly diminished in the Tmem45b deficiency neurons. Next, we used caffeine to activate RyR1 channel, inducing rapid internal Ca^2+^ release from the ER. Two rounds of caffeine stimulation were performed to assess the capacity of ER calcium store, including release and refilling (Figure 5H). Consistent with the thapsigargin results, the level of peak calcium signal was markedly reduced in cKO neurons (Figures 5I,J). After 20 min for ER calcium store refilling, a second caffeine stimulation was applied. The level of peak calcium signal was significantly reduced in cKO neurons, indicating Tmem45b deficiency impaired the ability of ER calcium refilling. Furthermore, to exclude the possible effects of gene cKO, we examine the ER calcium regulation on the cultured DRG neurons with acute knockdown of Tmem45b. Small interfering RNA, including Tmem45b siRNA (siTmem45b) or scramble siRNA, was transfected to the cultured DRG neurons (Supplementary Figure S8B). qPCR confirmed a significant reduction of Tmem45b expression in the siTmem45b treatment group (Supplementary Figure S8C). Calcium imaging analysis showed that the level of peak calcium signal induced by caffeine was significantly lower in siTmem45b-treated neurons (Supplementary Figures S8D,E). Both RYR and IP3R are expressed on the ER of DRG neurons. Next, we examined whether the Tmem45b deficiency affected IP3R-dependent calcium release. Previous reports showed that Trypsin could activate IP3R-mediated calcium release from the ER (Bartok et al., 2019). We examined the effect of Trypsin on dissociated DRG neurons under extracellular calcium-free conditions (Figure 5K). The results showed that Trypsin-induced calcium responses were significantly reduced in the Tmem45b cKO group compared with the WT group (Figures 5L–N). This finding indicated that IP3R-dependent calcium release was also impaired, which corresponded with the dyregulation of ER calcium storage. In summary, these data demonstrated that Tmem45b deficiency impaired the calcium regulatory capacity of the ER, affecting both calcium release and refilling.

**FIGURE 6:**
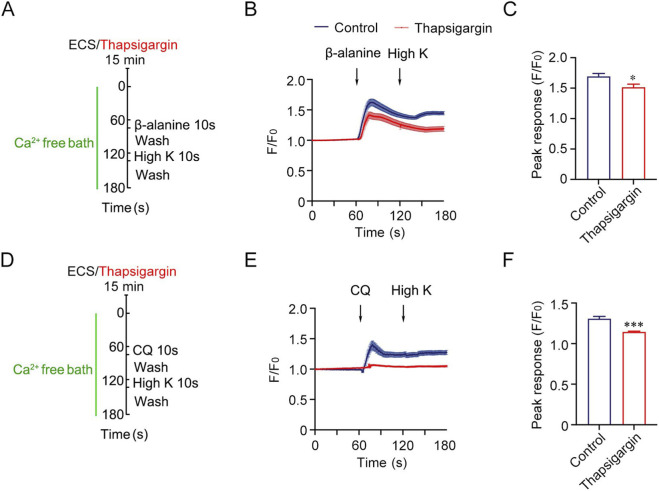
Inhibition of Serca reduces β-alanine- and CQ-induced intracellular calcium responses in DRG neurons. **(A,D)** Schematic illustration of experimental design using thapsigargin to inhibit Serca and deplete intracellular calcium stores. **(B)** Representative traces of β-alanine-induced calcium responses in DRG neurons with thapsigargin pretreatment (red) compared with control (blue). **(C)** Quantification of β-alanine-induced calcium responses (Control vs. thapsigargin, n = 170 vs. 158). **(E)** Representative traces of CQ-induced calcium responses in DRG neurons with thapsigargin pretreatment (red) compared with control (blue). **(F)** Quantification of CQ-induced calcium responses (Control vs. thapsigargin, n = 104 vs. 111). Two-tailed unpaired Student’s t test. DRG were collected from three mice each group. Data are expressed as the Mean ± S.E.M. *P < 0.05, ***P < 0.001.

To further assess the role of ER calcium dysregulation in β-alanine-induced calcium activity, we performed calcium imaging experiments under extracellular Ca^2+^ free condition (Supplementary Figure S9A). Under this condition, β-alanine-induced calcium release was significantly reduced in Tmem45b cKO neurons (Supplementary Figures S9B,C). Besides, CQ-induced calcium responses were also attenuated in Tmem45b cKO mice (Supplementary Figures S9D,E). This result is consistent with the reduced calcium responses to AITC observed in Tmem45b cKO mice (Figure 4H). To investigate the potential relationship between Serca1 dysfunction and β-alanine-induced calcium activity, we used thapsigargin, a pan-Serca inhibitor, to deplete intracellular calcium stores, thereby mimicking the reduced Calcium ER storage resulting from Serca1 downregulation. Calcium imaging under extracellular Ca^2+^ free condition revealed that thapsigargin treatment significantly attenuated β-alanine-induced intracellular calcium responses in DRG neurons (Figures 6A–C). Moreover, the calcium response triggered by CQ was almost completely abolished following thapsigargin treatment (Figures 6D–F). These results suggest that Serca activity is essential for the calcium transients evoked by both β-alanine and CQ, highlighting a potential role of Serca1 in mediating chronic itch signaling.

## Discussion

Our study demonstrated the novel role of Tmem45b in mediating nonhistaminergic itch. In Tmem45b cKO DRG neurons, calcium responses to β-alanine and AITC were significantly reduced. The loss of Tmem45b led to the downregulation of Serca1, disrupting ER calcium homeostasis and resulting in markedly reduced ER calcium release.

We confirmed that Tmem45b plays an important role in itch sensation, especially for chronic itch. Behavioral responses and calcium imaging confirmed a functional role of Tmem45b in Mrgprd^+^ neuron mediated itch pathways. Mrgprd^+^ neurons mediate nonhistaminergic itch via TRPA1 (Liu Q. et al., 2012; Wilson et al., 2011). Previous studies have implicated TRPA1 in chronic itch associated with atopic dermatitis (Yang et al., 2023), TRPA1-deficient mice exhibit reduced scratching behavior following AEW treatment (Kingwell, 2013). The inhibitory effect of Tmem45b loss on chronic itch may be attributed to reduced TRPA1-mediated calcium signaling, as evidenced by the decreased AITC-evoked calcium responses in Tmem45b deficient neurons. N-met LTC4 induces itch signals via Nppb peptides, which act on GRPR-positive neurons in the spinal cord (Mishra and Hoon, 2013; Sun and Chen, 2007). Previous research showed that block Nppb-GRPR signaling significantly reducing scratching behavior in atopic dermatitis model (Liu et al., 2020). We observed no difference between Tmem45b cKO mice and WT mice in the N-met LTC4-induced itch behaviors or calcium responses. Our data showed that Tmem45b expression was effectively ablated in Nppb^+^ neurons of cKO DRG (Figure 2D). Considering that Tmem45b deficiency impaired the DNFB-induced atopic dermatitis itch, Tmem45b might be involved in Nppb-mediated chronic itch rather than acute itch.

Calcium imaging showed Tmem45b deficiency specially impairs TRPA1, but not TRPV1. Under extracellular Ca^2+^ free condition, CQ-induced intracellular calcium responses were significantly reduced in Tmem45b cKO neurons. This indicates that Tmem45b is required for intracellular calcium release following CQ stimulation. However, under extracellular Ca^2+^ condition, CQ-induced calcium responses in Tmem45b cKO neurons were comparable to those in WT neurons. Even more surprisingly, Tmem45b cKO mice experienced stronger CQ-evoked itch compared to WT mice. To explore the underlying mechanisms, we employed RNAscope ISH under bright-field conditions, which offers ultra-high sensitivity capable of detecting individual RNA expression. Using this method, we observed a modest but detectable increase in Mrgpra3 expression in Tmem45b cKO mice. Upregulation of Mrgpra3 may compensate for impaired TRPA1 function and account for the unchanged CQ-induced calcium responses under extracellular Ca^2+^ conditions. Further investigation is needed.

Most unexpectedly, CQ-evoked scratching behavior was increased in Tmem45b cKO mice. CQ-induced acute itch peaks within 10–15 min after injection and diminishs as the compound is metabolized (Liu et al., 2009). Given the short duration and transient behavioral response, the enhanced itch phenotype observed in cKO mice is more likely due to peripheral sensitization mechanisms, potentially mediated by altered gene expression profiles. Upregulation of Mrgpra3 might partially explain this behavior phenotype. However, the key molecular invovled the signalling pathway underlying CQ-evoked itch is more important. scRNA-seq analysis revealed distinct *Trpa1* and *Trpv1* expression patterns in Mrgpra3^+^ and Mrgprd^+^neurons. *Trpv1* was more highly expressed in Mrgpra3^+^ neurons, whereas *Trpa1* showed higher expression in Mrgprd^+^ neurons (Supplementary Figure S6A). These findings indicate that CQ-induced itch via Mrgpra3 involves additional downstream pathways beyond TRPA1. Further analysis indicated that *Prkcq* (encodes PKCθ) is highly specific to Mrgprd^+^ neurons, which is a key regulator of T cell activation and NF-κB signaling, essential for Th2-mediated immunity (Marsland and Kopf, 2008). Adora2b and Kcnmb1 were highly expressed in Mrgpra3^+^ neurons (Supplementary Figure S6A). Adora2b, a Gs-coupled adenosine receptor, could promote cAMP accumulation and downstream PKA signaling, sensitize primary sensory neurons and amplify pain perception (Hu et al., 2016). In parallel, Kcnmb1, a modulatory β-subunit of BK (large-conductance Ca^2+^-activated K^+^) channels, activation of BK channels resulted in increased levels of intracellular calcium which might regulates neuronal excitability through calcium sensitivity (Scruggs et al., 2020). Although the exact functions of these molecules in itch perception is unclear, they may contribute to the distinct phenotypes of Mrgprd^+^ and Mrgpra3^+^ neurons. Further investigation is needed to clarify their functional relevance in itch modulation. Notably, we found Tmem45b expression remained detectable in Th^+^ neurons (Figure 2D). In contrast, Tmem45b was absent in Th^+^ neurons in the systemic knockout mice used previously (Tanioku et al., 2022). Th^+^ sympathetic neurons have been shown to promote inflammation during the early phase of collagen-induced arthritis (Pongratz and Straub, 2014). Thus, the phenotypic differences observed whthin the first 24 h after CFA injection may be due to Tmem45b expressed in Th^+^ neurons.

Our study suggested that Tmem45b was involved in regulating ER calcium homeostasis. *Atp2a1* (encoding Serca1) expression was significantly reduced in cKO DRG neurons, while *Atp2a2* and *Atp2a3* levels remained unchanged. Thus, the attenuated calcium responses to thapsigargin could be attributed to impaired Serca1 function. Our findings indicated that loss of Tmem45b downregulated Serca1, impairing the ER’s calcium release capacity and disrupting intracellular calcium balance. Under Ca^2+^ free condition, β-alanine-induced calcium flux was significantly reduced in the thapsigargin-treated group compared with control. This finding suggests that Tmem45b could regulate ER calcium homeostasis by modulating Serca1 expression, thereby influencing β-alanine-induced pruritic behavior. We also observed that thapsigargin treatment significantly attenuated CQ-induced intracellular calcium release although CQ-evoked itch behavior was markedly enhanced. While these results support a functional association between Tmem45b and Serca1, the causality remains indirectly inferred. Additional experiments, such as Serca1 re-expression or pharmacological rescue, is needed to definitively establish a direct mechanistic link. Nevertheless, our findings provide a strong foundation for future studies aimed at elucidating the precise molecular relationship between Tmem45b and ER calcium regulation.

Besides, we found that Tmem45b localized in the trans-Golgi network (TGN), which was responsible for the modification, sorting, and packaging of proteins and lipids in eukaryotic cells, including ion channels and GPCRs, such as Nav1.8 and δ Opioid Receptors (Cusdin et al., 2008; Guo et al., 2014). Protein localization in the TGN could regulate the ER-related gene expressions through ER stress and the unfolded protein response, such as TGN46 and Rab6 (Stenmark, 2009; Walter and Ron, 2011). Tmem45b may regulate Serca1 expression via a mechanism associated with the TGN. The underlying mechanisms remain to be elucidated. Finally, we demonstrate that Tmem45b is involved in the nonhistaminergic itch and the calcium regulation of ER, highlighting its importance in calcium homeostasis. These findings provide new insights into the mechanisms underlying the mechanical inflammation pain and nonhistaminergic itch, which could inform future therapeutic strategies.

## Data Availability

All data are available in the main text or the Supplementary Materials.” Transcriptomic data from this study has been submitted to the NCBI GEO database (accession number: GSE155622).
