# Supplementary material for: Tmem45b modulates itch via endoplasmic reticulum calcium regulation
Source: Front Physiol. 2025 Dec 10;16:1708686. doi: 10.3389/fphys.2025.1708686 (PMC12727599; doi:10.3389/fphys.2025.1708686)
Supplement: Supplementary file 1 [file DataSheet1.docx]

Supplementary Materials for

**Tmem45b Regulates itch via endoplasmic reticulum Calcium Regulation**

Sashuang Wang *et al.*

*Corresponding author. Chang-lin Li, Email: [licl@gdiist.cn](mailto:xxxxx@xxxx.xxx);

Wu-ping Sun, Email: wupingsun@email.szu.edu.cn

**This PDF file includes:**

Supplementary Text

Figs. S1 to S9

Tables S1

Supplementary Text

**Figure and table**

**Fig. S1.** **Characterization of peripheral and central terminals in Tmem45b cKO mice.**

(A) Immunofluorescence and (B) Western blot analysis show that Tmem45b antibody fails to detect a specific signal in the DRG of Tmem45b cKO mice following antigen pre-adsorption. Scale bar, 20 μm. (C–F) Immunofluorescence staining of Tuj1 (green) and CGRP (green) in both hairy and glabrous skin of Tmem45b cKO and WT mice. Scale bar, 50 μm. Quantitative analysis of the density of free nerve endings between control and cKO mice. (G–H) Immunostaining of IB4 (red) and CGRP (green) in the dorsal horn of the cervical and lumbar spinal cord. Scale bar, 200 μm. (I-L) Quantitative analysis of the central projections of non-peptidergic (IB4⁺) and peptidergic (CGRP⁺) sensory neurons in Tmem45b cKO mice compared to controls. Data are presented as mean ± SEM; n = 5 mice per group.

**Fig.S2. Gene expression analysis in the Tmem45b cKO mice.**

(A) Immunofluorescence statistical analysis of the percentage of Tmem45b-positive cells in homozygous mice. (B-M) qPCR results of cervical DRG. Columns represent the mean expression level of genes mRNA normalized to *Gapdh*. (N) Single-molecule RNAscope ISH of Mrgpra3 transcript at the single-cell level in individual DRG neurons. Scale bar, 20 μm. (O) Quantitative analysis of the intensity of Mrgpra3 transcript relative to the area of Mrgpra3^+^ neuron. (P) Dual-fluorescence RNAscope ISH showed the expression of Mrgpra3 (red) and Mrgprd (green) in Tmem45b cKO mice and WT mice. Scale bar, 20 μm. (Q) Quantitative analysis of the colocalization of Mrgpra3 with Mrgprd in DRG neurons. (R) The Venn diagram shows the distribution of DEGs in the DRGs of WT and cKO mice before and after CFA modeling. The red area represents DEGs in the WT group that are significantly differentially expressed before and after CFA modeling, while the blue area indicates DEGs in the cKO group. The purple area represents DEGs that are present in both groups. (S) (D) The figure presents the results of Gene Ontology Enrichment Analysis for DEGs in the WT and cKO groups before and after CFA modeling. For each gene set (genes uniquely present in the WT group, genes uniquely present in the cKO group, and genes present in both groups), the top 10 enriched pathways are displayed. The color of the points in the figure indicates the adjusted p-value (p.adjust), while the size of the points represents the GeneRatio value. Experiments are performed on more than 4 mice. Data are shown as means ± SEM. Two-tailed unpaired Student’s *t* test. ****P* < 0.001.

**Fig. S3 Sex-independent effects of β-alanine and CQ in Tmem45b cKO mice.**

(A, B) Behavioral responses to intradermal injection of β-alanine (1000 μg/50 μl), (WT vs. cKO, n = 7 vs. 8) and CQ (200 μg/50 μl), (WT vs. cKO, n = 6 vs. 6) on cheek. Data are shown as means ± SEM. Two-tailed unpaired Student’s *t* test. **P* < 0.05

**Fig. S4 Tmem45b is required for DNFB-induced chronic itch and AEW-induced epidermal changes.**

(A) Schematic diagram of DNFB model. (B) Behavioral analysis showed the scratching bouts of female Tmem45b cKO mice compared with WT mice in DNFB model (WT vs. cKO, n = 4 vs. 4). Statistical significance was assessed using two-way ANOVA test. (C) Experimental design for intrathecal injection of scramble or Tmem45b siRNA in male C57BL/6 mice. (D) qPCR results showed the knockdown efficiency. (E) Behavioral analysis revealed the scratching bouts of Tmem45b siRNA-treated mice on days 1, 3, and 5 after DNFB challenge compared to scramble controls (Scramble vs. siTmem45b, n = 9 vs. 12). Statistical significance was assessed using two-way ANOVA test. (F) Schematic diagram of DNFB model. (G) qPCR showed Tmem45b mRNA expression in DRGs after DNFB challenge. (H, I) Toluidine blue staining of skin sections collected on day 10 after DNFB treatment. Arrows indicate the mast cells in the basal dermis. Scale bar, 100 μm (J, K) H&E-stained skin sections from WT and Tmem45b cKO mice at AEW days 0, 2, 4, and 10. Scale bar, 100 μm. Data are shown as means ± SEM. Experiments are performed on more than 4 mice. Two-tailed unpaired Student’s *t* test. **P* < 0.05, ***P* < 0.01, ****P* < 0.001 #*P* < 0.05

**Fig. S5 The spinal glial cell activation was inhibited in the Tmem45b cKO mice.**

(A) The immunostaining result of GFAP in the cervical dorsal horn (green signal within the white dashed line). (B-C) Quanticification of the area ratio and total GFAP signal. (D) The immunostaining result of IBA1 in the cervical dorsal horn (red signal within the white dashed line). (E-F) Quanticification of the area ratio and total IBA1 signal. (G-J) qPCR result of marker genes representing the astrocyte (G and H) and microglia (I and J). Experiments were performed on more than 4 mice. Two-tailed unpaired Student’s t test. *P < 0.05, **P < 0.01, ***P < 0.001

**Fig. S6. Molecular complexity underlying itch signaling mediated by Mrgpra3⁺ and Mrgprd⁺ neurons.**

(A) The stacked violin plot illustrates the differential gene expression in the single-cell RNA sequencing data of adult mouse DRGs. Different colors represent different types of neurons in the adult mouse DRGs. (B) The heatmap shows the differential KEGG pathway. Red indicates high enrichment, while blue indicates low enrichment.

**Fig. S7. The subcellular location of Tmem45b.**

(A) The top triangle represents the sucrose concentration gradient, ranging from 20% to 60% in a continuous gradient. Immunoblotting of equal-volume aliquots of the supernatant fractions from mouse DRG showed that Tmem45b was present in ER and Golgi-containing fractions. The Western blot results were replicated in three independent experiments. (B-E) The immunocytochemistry results showed the expression of GFP (C), Calnexin (ER marker), (D) GM130 (cis-Golgi membrane marker), and (E) the Mito-tracer (mitochondrial marker) in COS7 cell. Scale bar, 10 μm.

**Fig. S8. The acute knockdown of Tmem45b impaired the calcium regulation of ER.**

(A) qPCR analysis shows the expression of *Atp2a1*, *Atp2a2*, and *Atp2a3*. Relative expression was normalized to the mean value of the WT group. (B) The flowchart represents the procedure for detecting calcium activity responses to cultured DRG neurons with Tmem45b siRNA or scramble siRNA transfected. (C) qPCR result showed the knockdown efficiency of Tmem45b siRNA. (D) Representative calcium images and (E) statistical analysis of the calcium release from the ER (WT vs cKO, n = 24 vs 24). DRG were collected from more than 4 mice. Two-tailed unpaired Student’s *t* test. **P* < 0.05, ***P* < 0.01, ****P* < 0.001

**Fig. S9 β-alanine- and CQ-induced intracellular calcium responses were reduced in Tmem45b cKO DRG neurons under calcium-free extracellular conditions.**

(A) Schematic of calcium imaging under calcium-free extracellular conditions. (B) Representative traces of β-alanine-induced calcium responses in DRG neurons from WT and Tmem45b cKO mice. (C) Quantification of the β-alanine-induced calcium flux peak. (B) Representative traces of CQ-induced calcium responses in DRG neurons from WT and Tmem45b cKO mice. (C) Quantification of the CQ-induced calcium flux peak. Data are shown as means ± SEM. DRG are collected from more than 4 mice. Two-tailed unpaired Student’s *t* test. **P* < 0.05, ****P* < 0.001

**Table1. Sequences of primers used in this study**

| **Gene** | **Forward primer** | **Reverse primer** |
| --- | --- | --- |
| Tmem45b genotyping | ACGCATGGTACAGGCTTGCA | GCTGCCATAGAGTGGGAACC |
| Tmem45b qPCR | GTCCTGTTCCCGCCATTT | ACTCCTCATCCGAGCCAC |
| Gapdh qPCR | CCAGCCTCGTCCCGTAGACA | CGCTCCTGGAAGATGGTGAT |
| Trpv1 qPCR | GCGAGTTCAAAGACCCAGAG | ACATCTGCTCCATTCTCCAC |
| Trpm8 qPCR | CAGCGGTACTTCCTGGTGCA | TTGTCGTTGGCTTTCGTGTT |
| Nppb qPCR | AAGTCCTAGCCAGTCTCCA | CTATCTTGTGCCCAAAGC |
| Il31ra qPCR | CTTATACTGTTTGGGTCATG | TACTTTCAGCAGGGTTGG |
| Mrgprd qPCR | TGGCATCCCAACAAACAC | CACATCCACCCAGTAGAGTAAG |
| Mrgpra3 qPCR | CTCAAGTTTACCCTACCCAAAGG | CCGCAGAAATAACCATCCAGAA |
| Th qPCR | TTCTGGAACGGTACTGTGGC | TCGGGTGAGTGCATAGGTGA |
| Trpa1 qPCR | GTCCAGGGCGTTGTCTATCG | CGTGATGCAGAGGACAGAGAT |
| Atp2a1 qPCR | GGAATGCAGAGAACGCTATCG | TCCTTTGCACTGACTTTCGGT |
| Atp2a2 qPCR | TGGAACAACCCGGTAAAGAGT | CACCAGGGGCATAATGAGCAG |
| Atp2a3 qPCR | GGAGCAGTTTGAGGACCTCTT | GGCCACGAGAATTAGCATGATG |
| Gal qPCR | CAGTAAGCGACCATCCAG | ATCCCAAGTCCCAGAGTG |
| S100b qPCR | TGGTTGCCCTCATTGATGTCT | CCCATCCCCATCTTCGTCC |
| Calca qPCR | TTTGAGGTCAATCTTGGAAAGCA | CTGAGCAGTGACACTAGAGCC |
| Ntrk1 qPCR | GCCTAACCATCGTGAAGAGTG | CCAACGCATTGGAGGACAGAT |
| Ntrk2 qPCR | GTTGACCCGGAGAACATCACG | ACTTTAAGCCGGAATCCACAAT |
| Ntrk3 qPCR | TCTTTGCCCAGCCAAGTGTAG | TCCTTGAGATGTCCGTAATGTTG |
| Aldh1l1 qPCR | CAGGAGGTTTACTGCCAGCTA | CACGTTGAGTTCTGCACCCA |
| Gfap qPCR | CCCTGGCTCGTGTGGATTT | GACCGATACCACTCCTCTGTC |
| Csf1r qPCR | TGCTAAGTGCTCTAGCCGAG | CCCCCAACAGTCAGCAAGAC |
| Cx3cr1 qPCR | GCCTCTGGTGGAGTCTGCGTG | CGCCCAAATAACAGGCCTCAGCA |
